# Genomic and phenotypic characterization of *Borrelia afzelii* BO23 and *Borrelia garinii* CIP 103362

**DOI:** 10.1371/journal.pone.0199641

**Published:** 2018-06-26

**Authors:** Sébastien Bontemps-Gallo, Kevin A. Lawrence, Crystal L. Richards, Frank C. Gherardini

**Affiliations:** Laboratory of Bacteriology, Rocky Mountain Laboratories, National Institute of Allergy and Infectious Diseases, National Institutes of Health, Hamilton, Montana, United States of America; University of Kentucky College of Medicine, UNITED STATES

## Abstract

In recent years, the number of Lyme disease or borreliosis cases in Eurasia has been dramatically increasing. This tick-borne disease is caused by *Borrelia burgdorferi* sensu lato, which includes *B*. *burgdorferi* sensu stricto, the main species found in North America, and *B*. *afzelii* and *B*. *garinii*, which are primarily responsible for the disease in Eurasia. Currently, research on Lyme disease has focused mainly on *B*. *burgdorferi* while *B*. *afzelii* and *B*. *garinii*, which cause disease with distinctly different symptoms, are less studied. The purpose of this study is to evaluate *B*. *afzelii* BO23 and *B*. *garinii* CIP 103362 as model organisms to study Eurasian Lyme disease. To begin our analyses, we sequenced, annotated the chromosomes of both species and compared them to *B*. *burgdorferi* strain B31. We also assayed shuttle vector, pBSV2, for transformation efficacy and demonstrated that these strains can be cultured on solid media. In addition, we characterized how physicochemical parameters (e.g., oxygen, osmolarity, oxidative stress) affect both growth and motility of the bacteria. Finally, we describe each strain’s antibiotic susceptibility and accessed their ability to infect mice. In conclusion, *B*. *afzelii* BO23 was more practical for *in vitro* and *in vivo* studies than *B*. *garinii* CIP 103362.

## Introduction

Lyme disease or borreliosis, caused by *Borrelia (Borreliella) burgdorferi sensu lato* (s.l.) [1], is transmitted to mammalian hosts through a bite of hard *Ixodes* ticks [2–5]. Lyme disease is the most common tick-borne disease with 300,000 estimated new cases in the United States of America [6, 7] and 85,000 detected new cases in Europe per year [8]. Climate change has expanded the geographic distribution of the vector leading to an increase of the number of cases [8, 9].

Thirty-five years ago, Dr. W. Burgdorfer and colleagues at the Rocky Mountain Laboratories (USA) identified the spirochete *B*. *burgdorferi* as the etiologic agent of Lyme disease [5] and were able to grow *Borrelia* spp. in a complex, chemical undefined rich medium (Barbour-Stoenner-Kelly, BSK) [10, 11]. Ten years later, Dr. G. Baranton and colleagues from the Institute Pasteur (France) demonstrated the genetic diversity in the Lyme disease-*Borrelia* related species [12]. Over the last 20 years, scientists and physicians have characterized distinct species belonging to the Lyme disease *Borrelia* and described related symptoms in more detail. In North America, only two species (*B*. *burgdorferi sensu stricto* (s.s.) and *B*. *mayonii*) have been described to cause disease in humans [3, 13]. In Eurasia, two species (*B*. *garinii* and *B*. *afzelii*) are the main cause of Lyme disease in humans [14, 15] while other species (*B*. *burgdorferi sensu stricto*, *B*. *spielmanii*, *B*. *bisettii*, *B*. *lusitaniae* and *B*. *valaisiana*) are occasionally found in patients suffering from borreliosis in Eurasia [2].

Symptoms can vary significantly from one species to another [16]. After transmission by infected ticks, an erythema migrans appears in 70–80% of the people infected by *B*. *burgdorferi s*.*s*. In contrast, only 20–30% of the people infected by *B*. *afzelii* or *B*. *garinii* develop this characteristic rash. All patients will develop flu-like symptoms, regardless of the causative species. During the early stage of disease, bacteria primarily localize at the bite site. As the disease progresses, bacteria disseminate to the skin and other tissues, including heart and central nervous system. About 15% of untreated people develop acute Lyme disease or a neuroborreliosis. Finally, in the late disseminated infection, bacterial spread throughout the whole body can lead to an Acrodermatitis Chronica Atrophins (ACA, *B*. *afzelii* infection), treatment resistant arthritis (*B*. *burgdorferi s*.*s*. infection), muscle weakness, cardiomyopathy or gastritis [2, 15, 16].

The genomes of *Borrelia* species are intriguing as they consist of a linear chromosome (about 910 kb) as well as of a set of circular and linear plasmids (10 circulars and 12 linears in *B*. *burgdorferi* B31 strain) [17, 18]. The number of plasmids varies depending on the species and seems to be affected by frequent reorganization [18–22]. The linear chromosome harbors the majority of essential genes involved in metabolism or regulation. Only a small set of genes encoding proteins that are required for growth localize to plasmids [18, 23]. Furthermore, surface proteins involved in virulence are mainly expressed from plasmids [4, 16, 24, 25]. Genetic tools have been developed to study gene function and regulation, making *Borrelia* species suitable for genetic studies to better understand biochemistry, physiology and virulence [26].

The increasing number of cases of Lyme disease in Eurasia has driven the scientific community to develop appropriate models to study and understand this disease. In this study, we investigated if *B*. *afzelii* strain BO23 and *B*. *garinii* strain CIP 103362 (both available from the ATCC strain collection) can be used as model organisms to study Eurasian Lyme disease. *B*. *afzelii* BO23 was isolated from human skin in Germany [27] while *B*. *garinii* CIP 103362 was isolated from an *Ixodes ricinus* tick in France [12]. Since genome sequence information is indispensable today, we first sequenced and annotated the genome of both strains. We also demonstrated that *B*. *afzelii* was transformable with the shuttle vector, pBSV2. We further studied the effects of physicochemical parameters encountered during the infectious cycle (oxygen, osmolarity, oxidative stress) on the growth as well as on the motility of these strains. Finally, we assayed the antibiotic susceptibility and infectivity of these two strains.

## Results

### Comparisons of the genome of the three strains

In modern biology, a strain has to satisfy several prerequisites to be considered a model organism. Among them, the availability of the genome sequence to study gene function and regulation is one of the most important. To establish if *B*. *afzelii* strain BO23 and *B*. *garinii* strain CIP 103362 can be used as model organisms for Eurasia Lyme disease, we first sequenced and annotated the genome of the two strains (Table 1).

**Table 1:** Comparison of the genome of the three *Borrelia* strains.

	*B*. *burgdorferi* B31				*B*. *afzelii* BO23				*B*. *garinii* CIP 103362			
	Accession #	Size (bp)	Gene	Protein	Accession #	Size (bp)	Gene	Protein	Accession #	Size (bp)	Gene	Protein
Chromosome	AE000783	910,724	860	797	CP018262	905,394	856	812	CP018744	905,638	856	808
Plasmids												
cp26	AE000792	26,498	26	24	CP018266	26,523	26	25	CP018750	27,038	26	25
cp32-1	AE001575	30,750	42	41								
cp32-3	AE001576	30,223	42	39								
cp32-4	AE001577	30,299	42	40								
cp32-6	AE001578	29,838	41	40								
cp32-7	AE001579	30,800	44	44								
cp32-8	AE001580	30,885	42	40								
cp32-9	AE001581	30,651	42	32								
cp9	AE000791	9,386	11	11	CP018274	8,898	10	10				
lp38	AE000787	38,829	36	28								
lp54	AE000790	53,657	65	55					CP018745	57,532	70	63
lp54-lp38 fusion					CP018263	85,180	97	85				
lp17	AE000793	16,821	19	14	CP018269	22,953	27	22	CP018751	20,915	20	19
lp21	AE001582	18,777	11	10								
lp25	AE000785	24,177	16	9								
lp28-1	AE000794	28,155	33	16								
lp28-2	AE000786	29,766	33	29								
lp28-3	AE000784	28,601	28	19	CP018265	27,055	27	19				
lp28-4	AE000789	27,323	25	19	CP018268	24,507	21	17				
lp28-7					CP018267	25,591	30	26	CP018749	27,075	31	28
lp28-8					CP018264	28,972	29	25				
lp36	AE000788	36,849	37	28					CP018746	37,053	33	21
lp5	AE001583	27,055	6	6								
lp56	AE001584	52,971	67	50								
Unassigned						104,625	142	124		80,859	115	82
total		1,543,035	1,568	1,391		1,259,698	1,265	1,165		1,156,110	1,151	1,046

The final assembled genome of *B*. *afzelii* consists of 32 contigs, including 1 linear chromosome (905,394 bp) and 31 contigs with lengths ranging from 1,117 to 85,180 bp. The final assembled genome of *B*. *garinii* consists of 12 contigs, including 1 linear chromosome (905,638 bp) and 11 contigs with lengths ranging from 1,085 to 57,532 bp.

Chromosomes of all strains were about the same size with an average 28.4% GC content (Table 1). Interestingly, less protein-encoding genes (797) are predicted for the chromosome of *B*. *burgdorferi* compared to *B*. *afzelii* (812) and *B*. *garinii* (808). We decided to look more closely at this difference (S1 Table). The three chromosomes shared 783 common protein-encoding genes. Eighteen genes were specific to *B*. *afzelii* and *B*. *garinii*. Furthermore, each chromosome had unique genes: 6 in *B*. *burgdorferi*, 7 in *B*. *afzelii* and 3 in *B*. *garinii* (S1 Table). Based on the annotation of the respective genes, none of these differences seems to provide new metabolic activities. Interestingly, *B*. *garinii* CIP 103362 has lost the gene encoding for PdeA, a cyclic di-GMP phosphodiesterase while other strains of *B*. *garinii* (BgVIR, NMJW1, SZ or PBr) harbor the gene. In *B*. *burgdorferi*, PdeA and PdeB are required to degrade c-di-GMP (cyclic diguanylate) into 2 GMP (guanosine monophosphate) [28]. The c-di-GMP signaling pathway is essential to complete the infectious cycle. In *B*. *burgdorferi*, disruption of *pdeA* negatively affects both motility and the ability to establish an infection in mice. [29].

Recently, Casjens *et al*. named plasmids in the two strains based on the sequence of their paralogous protein family 32 (PFam32) ParA partition proteins [30]. A total of 6 linear and 2 circular plasmids were assigned for *B*. *afzelii* B023, while only 4 linear and 1 circular plasmids in *B*. *garinii* CIP 103362 (Table 1). Interestingly, *B*. *afzelii* had a larger plasmid of 85,180bp, which seems to be a fusion of lp54-lp38. Fusion of linear plasmids has previously been detected in *Borrelia* genomes [20, 31]. More investigation will be required to determine the exact number of plasmids as well as to confirm the fusion of the linear plasmids. Besides facilitating genetic studies on these two strains, this study provides new genomes for phylogenetic studies on *B*. *burgdorferi s*.*l*. strains. Indeed, fewer strains have been sequenced and annotated for *B*. *afzelii* (HLJ01 [32], Pko [19], Tom3107 [33], K78 [34] and ACA-1 [19]) or *B*. *garinii* (BgVir [35], NMJW1 [36], SZ, PBr [35] and Far04 [19]) than for *B*. *burgdorferi s*.*s*. (18 strains).

### *B*. *afzelii* can be transformed with the pBSV2 shuttle vector

To assay the transformability of each strain, we attempted to transform each with the shuttle vector pBSV2 [37]. Transformation experiments were performed multiple times on each strain. *B*. *afzelii* and *B*. *burgdorferi* B31 A3 were equally transformable while numerous efforts to transform *B*. *garinii* with this vector were unsuccessful. The absence of transformants could be the result of more stringent restriction-modification (RM) systems in *B*. *garinii* [38]. It is important to remember that initial attempts to transform *B*. *burgdorferi* during the development of genetic systems for this bacterium were difficult until more genetically malleable strains were isolated.

In *B*. *burgdorferi*, disruption of the RM system by inactivation of *bbe02*, a type I restriction endonuclease subunit R, and *bbq67*, an adenine specific DNA methyltransferase, increases transformation efficiency with pBSV2 [38, 39]. An additional type I restriction endonuclease subunit R (*bbh09*) is annotated in *B*. *burgdorferi* but its role is unclear. Genomes of *B*. *afzelii* BO23 and *B*. *garinii* CIP 103362 also harbor a type I restriction endonuclease subunit R: BLA33_04690 in *B*. *garinii* and BLA32_04945 in *B*. *afzelii* are homologs of *bbe02*, and BLA32_05350 in *B*. *afzelii*. Four types of RM systems have been described in bacteria [40] and homologs of these systems have not yet been identified in *borrelia spp*.

The transformation frequency (TF) of pBSV2 was 4.31 x 10^−5^ ± 0.12 x 10^−5^ for *B*. *afzelii* BO23 and 2.05 x 10^−4^ ± 0.85 x 10^−4^ for *B*. *burgdorferi* B31 A3 *bbe02*^-^ *bbq67*^-^ (see Methods). This result was comparable to those observed by Rego *et al*., [38], although transformation efficiency (TE) was lower in our study for both strains (0.07 ± 0.01 for *B*. *afzelii* and 1.04 ± 0.2 for *B*. *burgdorferi*) than previously reported [38]. These results may be explained by the high concentration of DNA used for the transformation (~70 μg per transformation) compared to ~20 μg used in the previous study. Strains survived at a similar rate to the transformation process (1.4% for *B*. *burgdorferi* B31, 1.1% for *B*. *afzelii* BO23 and 1.3% for *B*. *garinii* CIP 103362). This data suggests that *B*. *afzelii* BO23 may be more suitable for genetic study than *B*. *garinii*. More study will be required to optimize the transformation protocols for these isolates.

### *B*. *afzelii* and *B*. *garinii* grow at a similar rate to *B*. *burgdorferi* under anaerobic and microaerobic atmospheres

**Fig 1:**
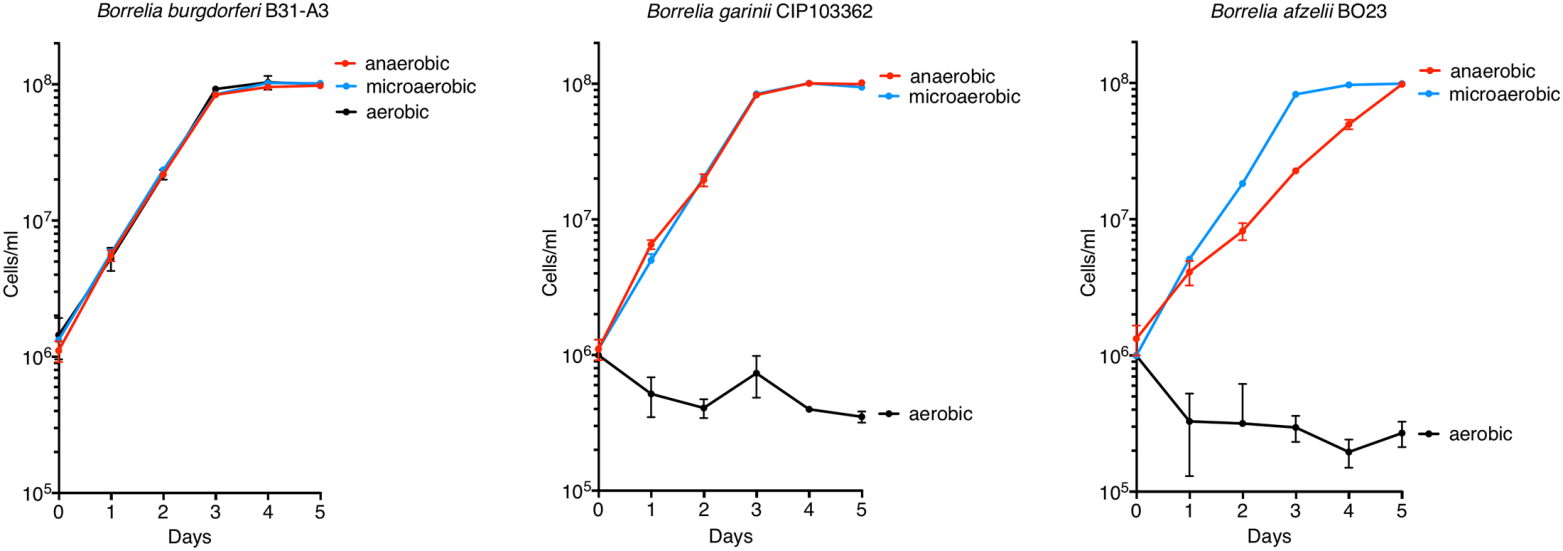
Effect of oxygen levels on growth rate of the 3 strains. Growth of *B*. *burgdorferi* B31, *B*. *afzelii* BO23 and *B*. *garinii* CIP 103362 in BSK-II medium under anaerobic (5% CO_2_, 5%H, 90%N_2_), microaerobic (3–5% O_2_, 5% CO_2_, 90%N_2+_) or aerobic (80% N_2_,20% O_2_, 0.05% CO_2_) conditions at 34°C. Data represent means ± standard deviation.

For a phenotypic characterization of the two strains, we first looked at the effects of different oxygen levels on the growth rate. Oxygen levels vary between the host and the vector [41] and within different tissues of the host [42, 43]. Furthermore, the oxygen level is also an important factor for *in vitro* culture. When we monitored the growth rate in BSK-II medium under different oxygen atmospheres, we were unable to observe growth of *B*. *afzelii* or *B*. *garinii* under aerobic conditions (Fig 1). However, aerobic conditions were not lethal to these strains since plating these aerobically-incubated cells on solid media under 3–5% O_2_,/5% CO_2_/90%N_2_ at 34°C indicated a >95% survival rate. *B*. *burgdorferi* and *B*. *garinii* grew normally under both anaerobic and microaerobic conditions with an average doubling time of 12 h, regardless of the strain or the oxygen level (Fig 1). *B*. *afzelii* grew normally under microaerobic conditions with a doubling time of 12 h. Interestingly, cell numbers started to decrease as cultures approached stationary phase (~1.1 10^8^ cell/ml). Under anaerobic conditions, *B*. *afzelii* grew slower than under microaerobic conditions with a doubling time of 19 h. Our data show that both strains can grow under microaerobic and anaerobic conditions but not under aerobic conditions. Why these strains are sensitive to aerobic growth conditions is not known.

### *B*. *afzelii* has more robust motility than *B*. *garinii* and *B*. *burgdorferi*

**Fig 2:**
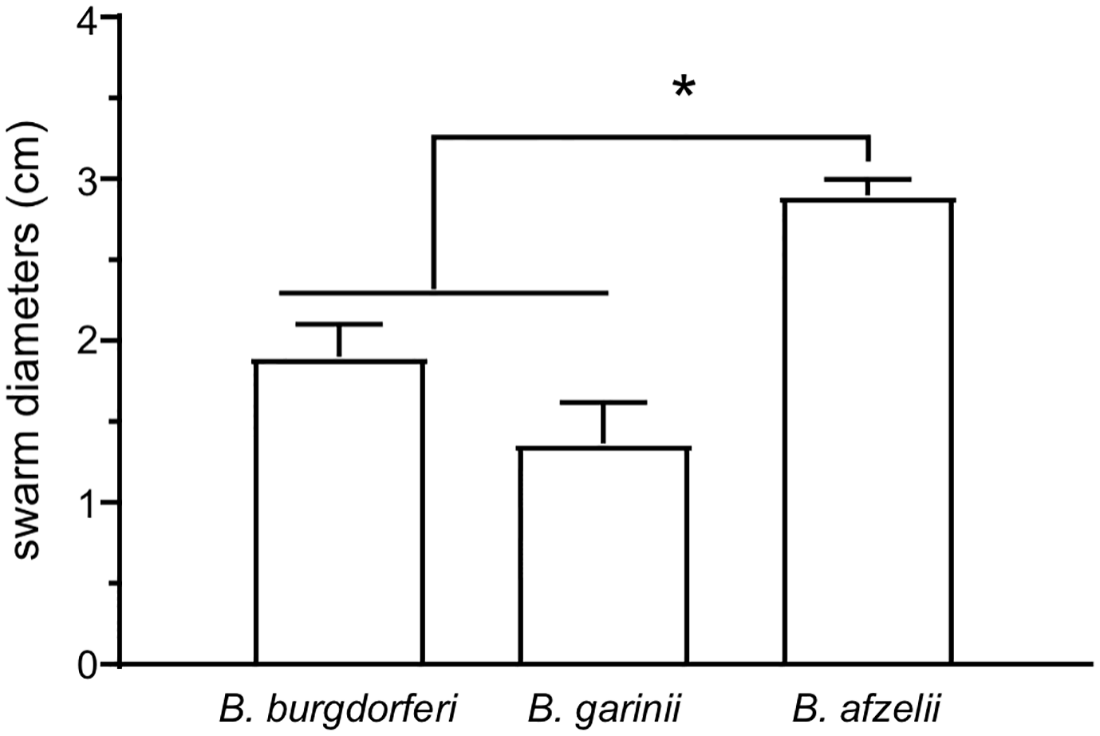
Motility of the three strains. Motility was measured in diluted BSK-II agar plates supplemented with 0.35% agarose. Swarm diameters were measured after 4d of incubation at 34°C in microaerobic conditions. Results reported are the average of three independent experiments. Data represent means ± standard deviation. An asterisk indicates a significant difference using a one-way ANOVA where p < 0.05.

Motility is one of the main virulence factors in bacteria [44, 45]. *Borrelia* species have distinctive endoplasmic flagella that enable infectious strains to disseminate throughout the whole body while non-motile mutants are avirulent [46]. To determine if there was a difference in the motility of the three *Borrelia* species, we performed a swarm plate assay as described previously by Motaleb *et al*. [47]. The strains were spotted in the swarm plate, grown for 4d and the swarm diameter was measured (Fig 2). *B*. *burgdorferi* B31 (1.9 +/- 0.2 cm) and *B*. *garinii* CIP 103362 (1.4 +/- 0.25 cm) displayed a similar motility (Fig 2). Interestingly, *B*. *afzelii* BO23 (2.9 +/- 0.1 cm) appeared to be more motile than the other strains that were tested. It is not clear whether this difference may provide *B*. *afzelii* BO23 any selective advantage for virulence or during transmission.

### *B*. *garinii* and *B*. *afzelii* are more sensitive to gentamicin and rifaximin than *B*. *burgdorferi*

Susceptibility to antibiotics is an important characteristic for both genetic manipulations and clinical treatment. We determined the susceptibility to different antibiotics by measuring the minimum inhibitory concentration (MIC) for the three strains. The MIC is defined as the lowest concentration of antibiotics at which the number of countable cells after 72h of incubation at 34°C does not exceed the initial number of cells. To determine the MIC, 5 x 10^6^ cells/ml were inoculated with increasing concentration of antibiotics. The final antibiotic concentration ranged from 0.015 to 32 μg/ml. We assayed 10 antibiotics targeting: cell wall synthesis (ampicillin, carbenicillin), RNA synthesis (rifaximin) and protein synthesis (chloramphenicol, doxycycline, gentamicin, kanamycin, spectinomycin, streptomycin, vancomycin) (Table 2). *B*. *garinii* was more sensitive to carbenicillin (0.015μg/ml vs 0.03 μg/ml), doxycycline (0.03 μg/ml vs 0.06 μg/ml) and spectinomycin (0.12 μg/ml vs 0.25 μg/ml) than *B*. *afzelii* and *B*. *burgdorferi*. *B*. *afzelii* and *B*. *garinii* were more sensitive to gentamicin (0.06 μg/ml vs 0.12 μg/ml) or rifaximin (8 μg/ml vs 16 μg/ml) than *B*. *burgdorferi*. No difference in the MIC between the three strains was observed for ampicillin (0.03 μg/ml), chloramphenicol (1 μg/ml), kanamycin (4 μg/ml), streptomycin (4 μg/ml) and vancomycin (0.25 μg/ml). Taken together, our data showed minor differences in the antibiotic susceptibility between the three strains with no relevant bearing on genetic engineering studies or on potential clinical treatment.

**Table 2:** Antibiotic susceptibility of *B*. *burgdorferi*, *B*. *afzelii* and *B*. *garinii*.

MIC (μg/ml)	*B*. *burgdorferi* B31-A3	*B*. *afzelii* BO23	*B*. *garinii* CIP 103362
Cell wall antibiotics			
Ampicillin	0.03	0.03	0.03
Carbenicillin	0.03	0.03	0.015
Protein synthesis antibiotics			
Chloramphenicol	1	1	1
Doxycycline	0.06	0.06	0.03
Gentamicin	0.12	0.06	0.06
Kanamycin	4	4	4
Spectinomycin	0.25	0.25	0.12
Streptomycin	4	4	4
Vancomycin	0.25	0.25	0.25
RNA synthesis antibiotics			
Rifaximin	16	8	8

Highlighted in red: the difference in antibiotic susceptibility compared to *B*. *burgdorferi*.

### *B*. *garinii* is more sensitive to oxidative stress than *B*. *afzelii* and *B*. *burgdorferi*

**Fig 3:**
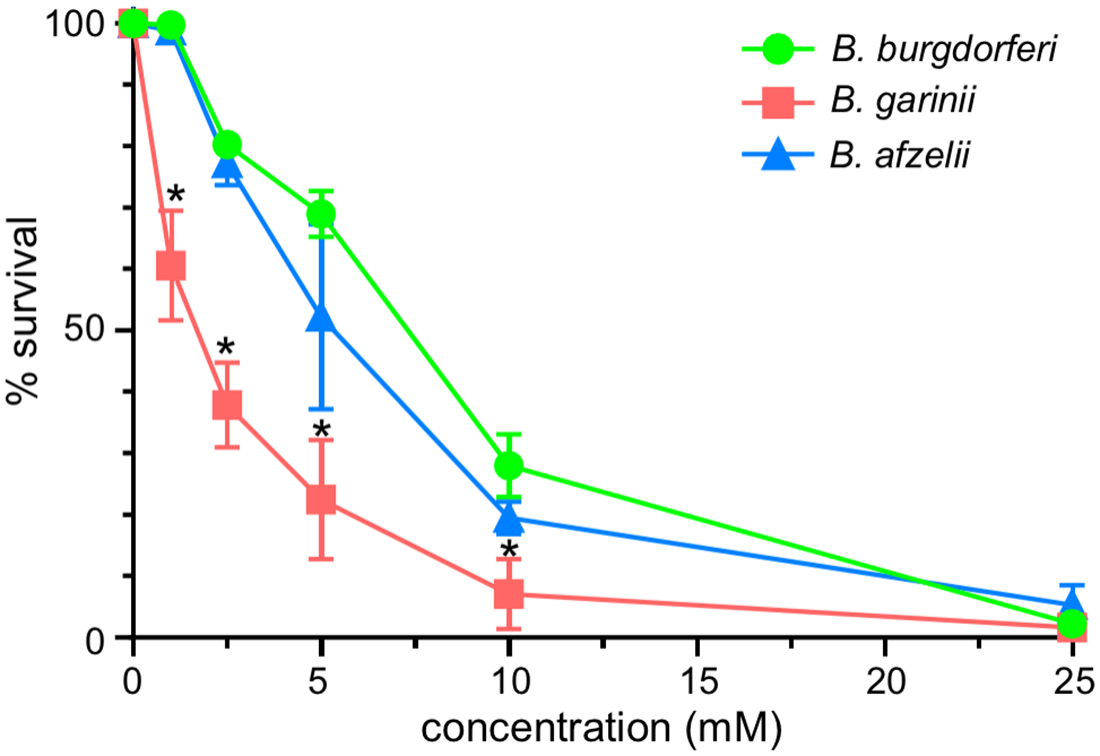
Survival to *tert*-butyl hydroperoxide. Early stationary phase cultures were incubated for 1.5h with 0, 1, 2.5, 5, 10 and 25 mM of *tert*-butyl hydroperoxide. Survival was determined by plating on BSK-II. Plates were incubated at 34°C under microaerobic condition for 7-14d to allow enumeration of CFUs. Untreated samples were used to standardize survival (100%). Data represent means ± standard deviation. An asterisk indicates a significant difference compared to *B*. *burgdorfe*ri B31 using a one-way ANOVA where p < 0.05.

During tick feeding, the *Borrelia* are challenged by reactive oxygen species (ROS) during the digestion of the blood meal [48]. Boylan *et al*. demonstrate that *B*. *burgdorferi* membrane lipids are the main targets of ROS [49]. To assay the susceptibility of these strains to ROS, we analyzed the survival of each strain at 1, 2.5, 5, 10 and 25 mM *tert*-butyl hydroperoxide (Fig 3). *B*. *burgdorferi* was sensitive to *tert*-butyl hydroperoxide to a similar level as observed previously [49]. While *B*. *afzelii* had similar resistance to ROS as *B*. *burgdorferi*, *B*. *garinii* was more sensitive. This higher susceptibility to ROS might explain why *B*. *garinii* was inhibited under aerobic growth conditions. Under those conditions, more ROS would be produced than would be predicted to be generated in microaerobic or anaerobic growth media. Additionally, sensitivity to ROS could affect survival in the host in response to a *Borrelia*-induced oxidative burst [50–54].

### *B*. *afzelii* has a narrower range of osmotolerance than *B*. *garinii* and *B*. *burgdorferi*

**Fig 4:**
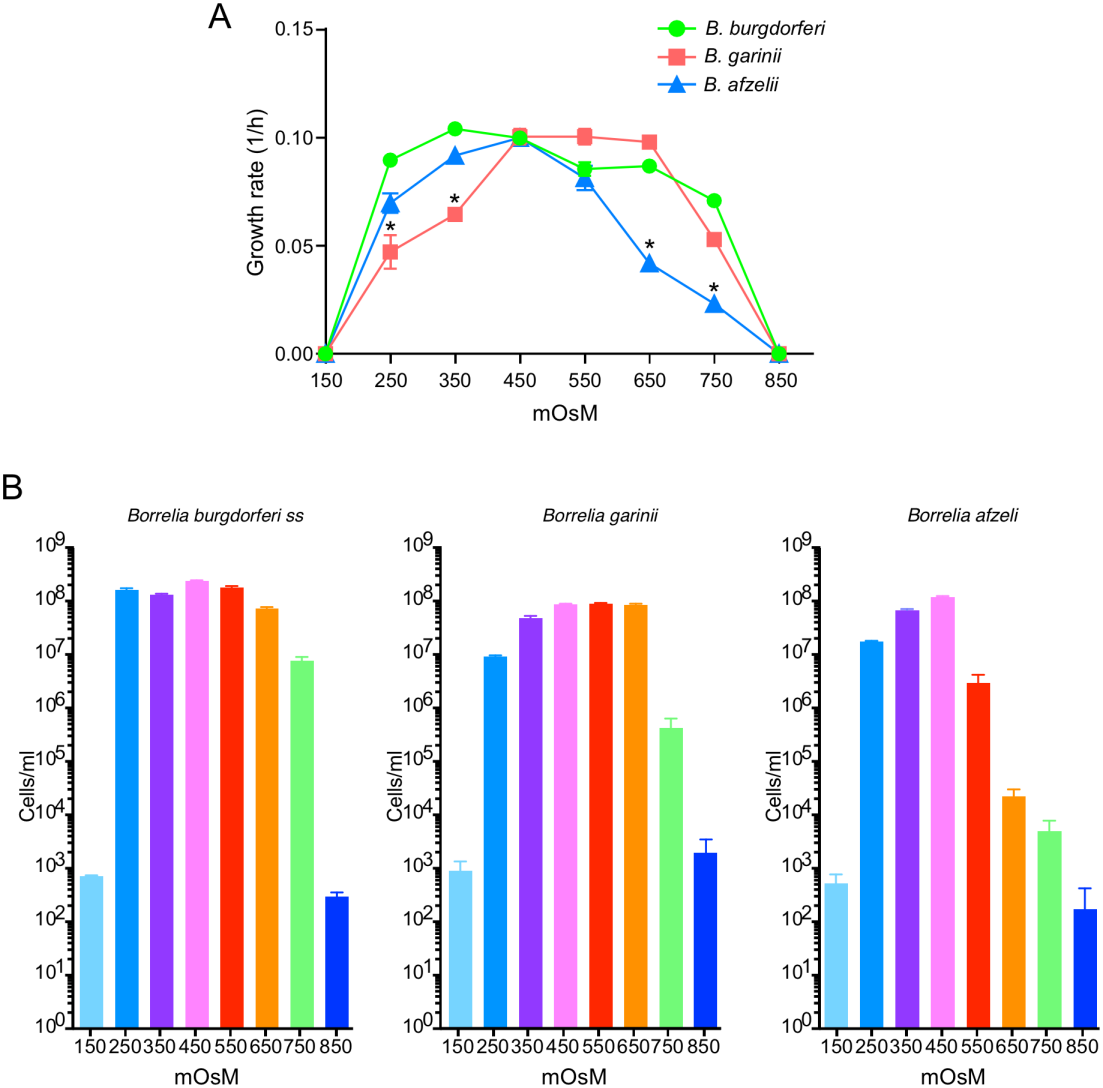
Rate of growth of *B*. *burgdorferi* B31-A3, *B*. *garinii* CIP 103362 and *B*. *afzelii* BO23 at various osmolarities. (A) Growth curves of strains were performed in BSK-II at various osmolarities (150 to 850 mOsM) under microaerobic conditions. (B) Graph of final densities of strains at various osmolarities. Growth was monitored daily by plating on BSK-II. Asterisks indicate a significant difference compared to *B*. *burgdorfe*ri B31 using a one-way ANOVA where p < 0.05. Data represent means ± standard deviation.

Osmolarity fluctuates in the midgut of ticks during the feeding of *I*. *scapularis* [41, 55, 56], *I*. *ricinus* [57–59] and *D*. *andersonii* [60–62]. According to the current model, osmolarity fluctuates in different hosts and in different vector tissues before, during and after feeding. It varies from 300 mOsM in the mammalian host [41, 63] to 600 mOsM in the midgut of unfed or replete ticks [41] and oscillates from 300 to 500 mOsM in the midgut, saliva or in the hemolymph during the feeding [41, 56]. To define the range of osmotolerance, we monitored the growth rate of *Borrelia* sp. in various osmolarities (150 to 850 mOsM) by plating on BSK-II at 24h intervals (Fig 4). As shown previously [41], *B*. *burgdorferi* B31 grew normally between 250 and 550 mOsM. At 650 mOsM, the cultures did not reach 10^8^ cell/ml. At 750 mOsM, bacteria stopped growing after only a few cycles of cell division. Cells lysed when cultures were grown at 150 or 850 mOsM. *B*. *garinii* CIP 103362 grew normally between 350 and 650 mOsM and only reached a density of 10^7^ cells/ml at 250 mOsM. Outside of that range (150, 750 and 850 mOsM) *B*. *garinii* cells appeared to lyse. Similar to *B*. *garinii*, *B*. *afzelii* BO23 grew normally at low osmolarity (350 and 450 mOsM), but only reached 2 x 10^7^ cells/ml at 250 mOsM. Cells were unable to grow at 150 mOsM or over 650 mOsM and rapidly lysed. Taken together, these major differences in osmotolerance could impact the survival of bacteria in the tick and may reflect differences in the vectors more common to the range (Eurasia) of these pathogens.

### *B*. *afzelii* and *B*. *burgdorferi* can colonize and persist in mice while *B garini* is unable to sustain a long-term infection

**Fig 5:**
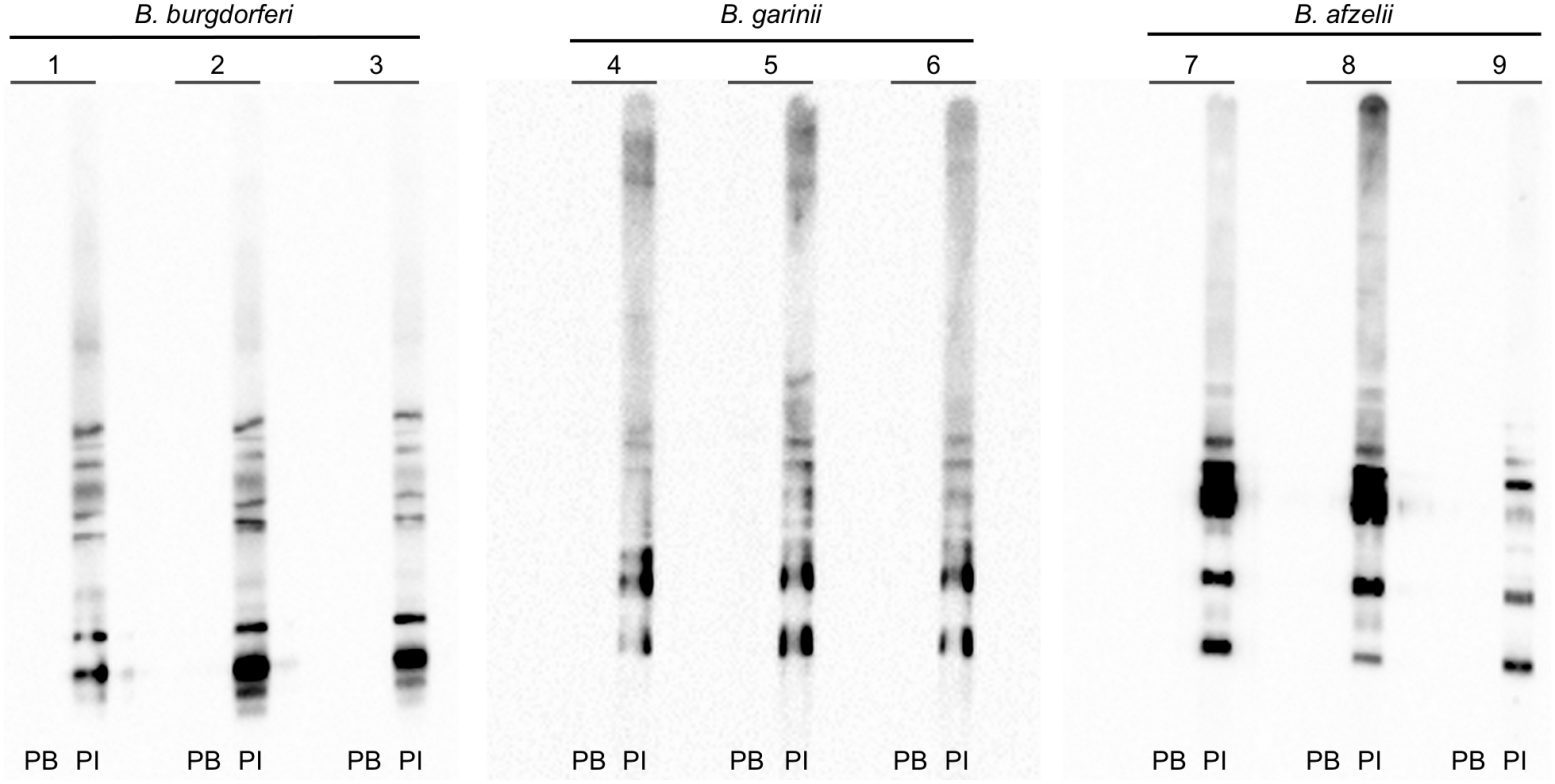
Immunoblot of *B*. *burgdorferi* B31-A3, *B*. *garinii* CIP 103362 and *B*. *afzelii* BO23 strains incubated with mouse sera. *Borrelia* strains were grown in BSK-II to mid-log phase and cell lysates were analyzed by immunoblotting. Cell lysates were incubated with serum obtained from mice either before (Pre-bleed, PB) or 6 weeks after needle inoculation (Post-infection, PI) with *B*. *burgdorferi* (lanes 1 to 3), *B*. *garinii* (lanes 4 to 6) and *B*. *afzelii* (lanes 7 to 9).

To determine if the strains were infectious in mice, naïve mice (3 per strain) were needle-inoculated with 10^6^CFU of *B*. *garinii* or *B*. *afzelii* grown to ~5 x 10^7^ cells/ml under a microaerobic atmosphere at 34°C. After six weeks, tissue samples (bladder, joint, skin, ear) were removed from each animal, cultured in BSK-II for 2 months and checked every 7 d by darkfield microscopy to determine the presence of bacteria (Table 3). After 2 wks, spirochetes were observed in cultures from tissues from *B*. *afzelii* and *B*. *burgdorferi* infected mice. *B*. *burgdorferi* were re-isolated from all cultured tissues while *B*. *afzelii* was re-isolated from skin, bladder, and ear tissues but never from joint tissue. *B*. *garinii* was not re-isolated from any tissues of infected mice. Interestingly, mice infected with all strains seroconverted after 6 wks (Fig 5) suggesting that *B*. *garinii* could produce an initial infection leading to an adaptive immune response but was unable to persist in the mouse. This finding led us to re-examine the genome of *B*. *garinii* with a focus on identifying genes which had been shown to affect virulence of *B*. *burgdorferi* in mice (e.g. *sodA*, *bosR*, *pdeA*, *pdeB*, etc,) [4, 24]. One notable difference was that the *B*. *garinii* CIP 103362 genome lacked the gene encoding a cyclic di-GMP phosphodiesterase (PdeA) which has been shown to affect virulence in *B*. *burgdorferi* [29]. Additionally, we have shown that this strain is more sensitive to ROS which also might affect its ability to infect experimental animals. Currently, we do not know the exact reason that this strain presented reduced virulence but, based upon these data, *B*. *afzelii* BO23 is more suitable for infectious studies than *B*. *garinii* CIP 103362.

**Table 3:** Mouse infectivity results of *B*. *burgdorferi*, *B*. *garinii* and *B*. *afzelii* strains.

Strain	Tissue cultures positive				Seroconversion
	Bladder	Joint	Skin	Ear	
*B*. *burgdorferi* B31-A3	+	+	+	+	+
	+	+	+	+	+
	+	+	+	+	+
*B*. *garinii* CIP 103362	-	-	-	-	+
	-	-	-	-	+
	-	-	-	-	+
*B*. *afzelii* BO23	+	-	+	-	+
	+	-	+	+	+
	-	-	+	+	+

## Discussion

The increasing number of cases of Lyme disease in Eurasia, an area of 5 billion human beings, should motivate the scientific community to find an appropriate bacterial model to study this disease. Publications of the last 30 years demonstrate that *B*. *afzelii* and *B*. *garinii* are the two-main species responsible for Lyme disease in Eurasia [2, 16]. Differences in clinical features are observed between the different species: arthritis for American species and acrodermatitis chronica atrophicans for Eurasian species [2, 3, 16]. Additionally, climate change expands the geographic distribution of the vector by allowing ticks to colonize new areas [64–67].

In this study, we investigated if *B*. *garinii* CIP 103362 and *B*. *afzelii* BO23 could be used to study Eurasian Lyme disease via a murine model. Both strains are available and maintained by the ATCC. We first sequenced and partially annotated the genome of both strains in order to make them usable for genetic and phylogenetic studies. Genomes were deposited in the NCBI database to be publicly available. Although the strains showed some minor differences in the numbers of protein-encoding genes (Table 1, S1 Table), none of these genes seem to impart new metabolic capabilities or virulence capacities that would offer a selective advantage in the natural infectious cycle.

Knowing the appropriate conditions to grow *Borrelia* species is a prerequisite to any investigation of changing physicochemical factors or virulence analyses. Previously, Veinović and colleagues showed that *B*. *afzelii* and *B*. *garinii* have an optimal growth temperature of 33°C [68]. Our initial studies of *in vitro* growth conditions of *B*. *afzelii* and *B*. *garinii* indicated that both species were unable to grow under aerobic conditions. Since, Lyme spirochetes do not metabolize oxygen, it seems likely that the sensitivity to high oxygen reflects a corresponding sensitivity to ROS. Indeed, this seemed to be the case for *B*. *garinii* which was more sensitive to ROS and aerobic growth conditions than *B*. *afzelii* or *B*. *burgdorferi*. It seems quite likely that these differences in aerotolerance and sensitivity to ROS, might reflect differences in the expression of genes encoding proteins which function to degrade ROS (e.g., SodA, CoADR, etc.) [4, 24]. Importantly, we demonstrated that both species prefer to grow under limited oxygen (microaerobic or anaerobic) and these growth conditions should facilitate *in vitro* studies of the spirochetes (Fig 1).

We further characterized the two strains by assessing their respective motilities. *B*. *garinii* CIP 103362 has the same capacity as *B*. *burgdorferi* B31-A3 (Table 4, Fig 2), while *B*. *afzelii* B023 was more motile. Initially, we believed that this feature would allow for a more efficient colonization of the host, compared to *B*. *burgdorferi*. Despite this observation, in our hands, we were unable to re-isolate these spirochetes from all the murine tissues tested (Fig 5, Table 3) which suggested that increased motility did not enhance virulence of *B*. *afzelii*. However, it is also possible that the infection by *B*. *afzelii* has already switched from a systemic infection to a localized infection after 6 wks [16]. Another explanation could be that *Borrelia* species are well known to have some organotropism [2, 3, 13, 16]. *B*. *afzelii* or *B*. *garinii* have been observed to reside in the skin or the nervous system while *B*. *burgdorferi* s.s. also invades the joints [2, 3, 16]. The initial hypothesis by Hubálek and colleagues was that a difference in the optimal growth temperature is the reason for this organotropism [69]. Recently, a study by Veinović and colleagues didn’t support this hypothesis [68]. Instead, they suggest that organotropism could be explained by the ability to grow at different oxygen levels. Indeed, the dissolved oxygen concentrations fluctuate in mammalian tissues [42, 43]. However, our data doesn’t support this hypothesis since the three strains grew similarly under microaerobic conditions (Fig 1).

**Table 4:** Summary of the results.

	*B*. *burgdorferi* B31-A3	*B*. *garinii* CIP 103362	*B*. *afzelii* BO23
Oxygen level^a^	Anaerobic ++ Microaerobic ++ Aerobic ++	Anaerobic ++ Microaerobic ++ Aerobic -	Anaerobic + Microaerobic ++ Aerobic -
Motility^b^	+	+	++
Antibiotic^c^		More sensitive to: Carbenicillin, Doxycycline, Gentamicin, Spectinomycin, Rifaximin	More sensitive to: Gentamicin, Rifaximin
ROS^d^ (50% survival)	*t*-BH: 5/10mM	*t*-BH: 1/2.5 mM	*t*-BH: 5 mM
Osmotolerance^e^	250–550 mOsM	350–650 mOsM	350–450 mOsM
Mouse infectivity^f^	+	-	+
Transformation^g^	+	-	+

^a^: Ability of *B*. *burgdorferi* to grow in aerobic conditions was reported previously [10].

^b^: Motility was defined based on *B*. *burgdorferi*: +: motile like *B*. *burgdorferi*, ++: more motile than *B*. *burgdorferi*.

^c^: Antibiotic susceptibility was defined based on *B*. *burgdorferi* susceptibility (see Table 2).

^d^: *t*-BH: *tert*-butyl hydroperoxide.

^e^: Psmotolerance was defined as ‘able to grow up to high density, *i*.*e*. 10^8^ cells/ml’.

^f^: Strain was considered infectious if bacteria were re-isolated from mouse tissue (see Table 3).

^g^: Cell was (+) or was not (-) transformed with the pBSV2 shuttle vector.

Since water flux and osmolarity fluctuate during the infectious cycle [41, 55–62], we also compared the osmotolerance of the three strains. All strains were able to grow at low osmolarity, *i*.*e*. blood-like osmolarity (Fig 4, Table 4). Interestingly, only *B*. *burgdorferi* and *B*. *garinii* were able to survive and grow at higher osmolarity, *i*.*e*. midgut osmolarity (Fig 4, Table 4). This result is of interest because Jacquet and colleagues showed recently that *B*. *afzelii* cell numbers dramatically decrease in *I*. *ricinus* [70] while *B*. *burgdorferi* numbers stayed constant between two feedings [71]. Sensitivity to osmolarity could explain, in part, this decrease in the number of *B*. *afzelii* in the midgut of the vector. As shown previously, the main effect of increased osmolarity is an increase in the pressure on the cell wall with corresponding inhibition of growth and/or cell death [72, 73]. However, Bourret and colleagues showed that specific mutations in genes encoding DNA repair enzymes in *B*. *burgdorferi* dramatically affect cell growth in the tick midgut but did not affect transmission [74]. This study demonstrated that robust growth and high spirochete loads are not an absolute requirement for successful transmission. Taken together, these data suggest that the transmission of *B*. *afzelii*, despite its sensitivity to “high” osmolarity, may not be incumbered by osmotic stress.

Finally, survival in the host is also dependent on the ability to survive and proliferate in the presence of the antagonistic host immune responses. Here, we showed that *B*. *garinii* CIP 103362 cannot be isolated from tissues of an infected mouse (Tables 3 and 4, Fig 5). The result was surprising since Xu and Johnson reported that the strain was virulent in hamsters at a high infective dose (10^8^ cells/animal) [27]. In our study, the strain was not able to establish a long-term infection but was able to trigger an adaptive immune response in mice (Fig 5) suggesting that the strain could possibly establish an acute infection but not persist in the mammalian host. The inability to colonize/persist in a mouse could also be the consequence of the loss of *pdeA* from the in *B*. *garinii* CIP 103362 genome. Indeed, a similar phenotype is observed in a *B*. *burgdorferi* B31-A3 *pdeA* mutant that could not be re-isolated from the tissue of an infected mouse but could trigger seroconversion when a high infective dose (10^7^ cells/mouse) was used [29]. Additionally, *Borrelia* species are known to induce an oxidative burst during an infection [50–54]. Based upon our analyses, it seems plausible that the sensitivity to ROS demonstrated by *B*. *garinii* CIP 103362 might also affect survivability in infected animals (Fig 3). As previously mentioned, the *B*. *garinii* genome harbors the genes encoding oxidative stress related proteins identified in *B*. *burgdorferi* [75, 76]. Finally, it has been shown in multiple studies that plasmid loss can dramatically impact the infectivity of Lyme and Eurasian Lyme disease spirochetes [27, 77]. Using our genome and plasmid sequences, Casjens *et al*. cataloged some of the paralogous plasmid groups from *B*. *garinii* but not all of the plasmids have been accounted for. So we cannot eliminate plasmid loss as a factor in the phenotype of this strain. Currently, we do not know which of these factors are contributing to the reduced virulence of observed for *B*. *garinii*.

In summary, our data, summarized in Table 4, demonstrates that *B*. *afzelii* BO23 is currently the most useful model organism to study protein function, gene regulation and the pathogenesis of Eurasian Lyme disease spirochetes. Furthermore, phenotypic differences between *B*. *afzelii* and *B*. *burgdorferi* provide a first step in understanding the observed clinical and ecological dissimilarities between these different forms of Lyme disease.

## Materials and methods

### Bacterial strains, media and growth conditions

The bacterial strains used in this study are *B*. *garinii* (CIP 103362 / 20047 / ATCC 51383), *B*. *afzelii* (BO23 / ATCC 51992), *B*. *burgdorferi* (B31-A3 [78], but also available at ATCC 35210 as B31). *Borrelia* strains were grown in Barbour-Stoenner-Kelly II medium (BSK-II) (Table 5). Cultivation was performed at 34°C under microaerobic condition (3% O_2_, 5% CO_2_), and when indicated, under anaerobic (5% CO_2_, 5%H_2_, balance N_2_) or aerobic (18% O_2_ in Hamilton, MT, USA) conditions. Cell densities were determined by dark-field microscopy. The osmolarity of the BSK-II medium is 450 mOsM. To generate high-osmolarity media, NaCl was added. To obtain low-osmolarity medium, ddH_2_O was added to dilute 1.8-fold the BSK-II. Low-osmolarity BSK-II media support normal growth [41]. Osmolarity of growth media (mOsM) was measured with a vapor pressure osmometer (model 3320, Advanced Instruments, Inc., MA, USA). Growth rates were defined during the exponential phase and defined as 1/doubling time and expressed in 1/h.

**Table 5:** BSK-II composition and reference, lot number of each ingredient.

Ingredients	Concentration	Reference	Lot #	Company
CMRL1066, 10X	9.8 g/L	C5900-05	L16122801	USBiological, Salem, MA, USA
Neopeptone	4 g/L	211681	6326665	BD Difco, Franklin Lakes, NJ, USA
Yeastolate	1.6 g/L	255772	5047809	BD Difco, Franklin Lakes, NJ, USA
Glucose	4 g/L	215530	6160813	BD Difco, Franklin Lakes, NJ, USA
Sodium bicarbonate	1.53 g/L	7412–12	144385	Macron Avantor, Center Valley, PA, USA
Sodium citrate dehydrate	0.56 g/L	6132-04-3	143314	Thermo Scientific, Rockford, IL, USA
Pyruvic acid sodium salt	0.64 g/L	102926	1638KA	MP Biochemical, Santa Ana, CA, USA
N-acetyl-D-glucosamine	0.32 g/L	A4106	099K0081	Millipore Sigma, St. Louis, MO, USA
Bovine Serum Albumin	40 g/L	821005	106	Millipore Sigma, St. Louis, MO, USA
HEPES sodium salt	6 g/L	391333	2805384	Millipore Sigma, St. Louis, MO, USA
Rabbit serum	6%	31125	17538 33036	Pel-Freez, Rogers, AR, USA

### Genome sequencing, assembling and annotation

The genomes of *B*. *afzelii* and *B*. *garinii* were sequenced at ACGT, Inc. (Wheeling, IL, USA) using Illumina MiSeq and the NextSeq 500 platform.

DNA was extracted by the Phenol/Chloroform method [79] from cells grown in BSK-II medium.

Sequencing was performed on the Illumina NGS platform with PE150 reads. Both paired-end and mate-pair libraries were used for the assembly. For *B*. *afzelii*, a total of 4,627,524 and 7,034,705 read pairs were generated from the paired-end (average 550 bp fragment size) and mate-pair libraries (average 600 bp fragment size), respectively. For *B*. *garinii*, a total of 20,718,626 and 4,487,254 read pairs were generated from the paired-end (average 550 bp fragment size) and mate-pair libraries (average 600 bp fragment size), respectively.

The final assembled genome of *B*. *afzelii* consists of 32 contigs, including 1 linear chromosome (905,394 bp) and 31 contigs with lengths ranging from 1,117 to 85,180 bp.

The final assembled genome of *B*. *garinii* consists of 12 contigs, including 1 linear chromosome (905,638 bp) and 11 contigs with lengths ranging from 1,085 to 57,532 bp.

Prediction of protein-coding sequences and annotation of the chromosome and plasmids were performed by the Prokaryotic Genome Annotation Pipeline v3.1 (http://www.ncbi.nlm.nih.gov/genome/annotation_prok/).

### Nucleotide sequence accession numbers

The genome sequence of *B*. *garinii* strain CIP 103362 has been deposited in the GenBank database under accession numbers: chromosome (CP018744) and plasmids (cp26: CP018750, cp32 partial: CP018754 and CP018755, lp28-7: CP018749, lp54: CP018745, unassigned: CP018746 to CP018748 and CP018751 to CP018753)

The genome sequence for *B*. *afzelii* strain BO23 has been deposited in the GenBank database under accession numbers: chromosome (CP018262), and plasmids (cp26: CP018266, lp28-3: CP018265, lp54-lp38 fusion: /CP018263, unassigned: CP018264, CP018267 to CP018293).

### Transformation of pBSV2 in *Borrelia spp*.

pBSV2 [37] was transformed into *B*. *burgdorferi* B31-68-LS [80], *B*. *afzelii* BO23 and *B*. *garinii* CIP 103362 as described previously by Samuels, D.S. [81].

To prepare competent cells, bacteria were grown in BSK-II to a density of 5 x 10^7^ cells/mL. Cells were centrifuged at 9,600xg, 10 min at 20°C and resuspended in 0.25 volumes of ice-cold EPS (93g /L of sucrose, 15% Glycerol). Cells were pelleted again at 7,700xg, 10 min, 10°C and resuspended in 0.1 volume of ice-cold EPS. Cells were centrifuged at 7,700g, 10 min, 10°C and resuspended in 0.0025 volume ice-cold EPS. Cells were transferred into a microtube. Cells were examined by darkfield microscopy for a suspension without clumping of bacteria.

To transform the cells, 70μg of purified vector DNA were mixed with 100 μL of freshly prepared competent cells. The mixture was transferred to an electroporation cuvette and cells were transformed by electroporation using a Gene Pulser Xcell Electroporator (Bio-Rad, Hercules, CA, USA) using the following settings: 2.5 kV, 25 μF, and 200 Ω. Then, cells were resuspended in 5 mL of warm BSK-II. Prior to plating, cells were allowed to recover for 12h at 34°C under microaerobic conditions. A no antibiotic control plate was performed to assess viability of the spirochetes after transformation.

To plate the cells, BSK-II containing 0.68% Ultrapure^™^ agarose (16500500, Thermo Scientific, Rockford, IL, USA) was warmed-up to 55°C and 200 μg/ml kanamycin (1355006, Millipore Sigma, St. Louis, MO, USA) was added to the medium. As a bottom layer, 20 ml of medium was poured in the Petri dish. When the bottom plate was solidified, 30 ml of BSK-II with antibiotic were poured containing 1 ml of the transformation. Plates were then incubated at 34°C under microaerobic conditions. Transformants appeared after 7 to 10 d of incubation.

Kanamycin-resistant colonies were analyzed by PCR to confirm the construction using Kan-F (CGAGGCCGCGATTAAATTCC) and Kan-R (AGCCGTTTCTGTAATGAAGGAGA) as primers.

### Transformation frequency and efficiency

Transformation frequency (TF) and transformation efficiency (TE) were calculated as described previously by Rego *et al*. [38].

### Motility assay

The motility assay was performed as described before by Motaleb and colleagues [47]. Swarm plate assays were tested on BSK-II medium diluted 1:10 in PBS plates containing 0.35% agarose (16500–500, Thermo Scientific, Rockford, IL, USA). Cells were washed twice in HN buffer (50 mM HEPES pH 7.5, 50 mM NaCl) and resuspended in BSK-II. 5 x 10^5^ cells in 5 μl were spotted onto plates containing BSK-II medium diluted 1:10 in PBS without divalent cations (21-040-CV, Corning, Corning, NY, USA). Plates were incubated 4 d at 34°C under microaerobic conditions. Swarm diameters were measured in centimeters.

### Statistical analysis

Prism 7 software (GraphPad Software, Inc.,La Jolla, CA, USA) was used to analyze data using a One-Way ANOVA with a Geisser-Greenhouse correction; a value of p < 0.05 was considered significant.

### Antibiotic susceptibility

Antibiotic susceptibility was performed as described previously by Sicklinger and colleagues [82]. Briefly, antibiotics (Table 6) were dissolved in warm BSK-II medium, pH 7.6, and supplemented with 6% Rabbit Serum. The final concentration of antibiotics ranged from 0.015 to 32 μg/ml. To determine the MIC, the initial cell density was adjusted to 5 x 10^6^ cells/ml. After 72h of incubation at 34°C, cells were counted again.

**Table 6:** Antibiotics used in this study.

Antibiotic	Reference	Company
Ampicillin	A1593	Millipore Sigma, St. Louis, MO, USA
Carbenicillin	C9231	Millipore Sigma, St. Louis, MO, USA
Chloramphenical	C0857	Millipore Sigma, St. Louis, MO, USA
Doxycycline	67457-437-10	Mylan, Canonsburg, PA, USA
Gentamicin	G1264	Millipore Sigma, St. Louis, MO, USA
Kanamycin	1355006	Millipore Sigma, St. Louis, MO, USA
Spectinomycin	PHR1441	Millipore Sigma, St. Louis, MO, USA
Streptomycin	S9137	Millipore Sigma, St. Louis, MO, USA
Vancomycin	V2002	Millipore Sigma, St. Louis, MO, USA
Rifaximin	Y0001074	Millipore Sigma, St. Louis, MO, USA

### ROS susceptibility assays

Susceptibility to ROS was performed as described previously [49]. Strains were grown in BSK-II medium under microaerobic conditions at 34°C starting at 1 x 10^5^ cells/ml to early stationary phase of growth at 5 x 10^7^ cells/ml. Cells were exposed to the indicated concentration of Luperox^®^ TBH70X, *tert*-Butyl hydroperoxide (458139, Millipore Sigma, St. Louis, MO, USA) for 1.5h in BSK-II. Samples were plated on BSK-II and incubated at 34°C under microaerobic conditions for 7-14d to allow enumeration of CFUs.

### Immunoblots

For analysis of cell lysates by Western blot, bacteria were grown to mid-log phase at 34°C under microaerobic conditions. The cells were harvested by centrifugation, washed twice in HN buffer (50 mM HEPES pH7.5, 50 mM NaCl), resuspended in 0.25M Tris-HCl pH 6.8 and lysed by sonication. The protein concentration was determined with Take3 micro-volume plates in a Synergy 2 Multi-Mode plate reader (BioTek Instruments, Winooski, VT, USA). 40 μg of protein was loaded in a 4–15% pre-cast SDS-PAGE gel (Bio-Rad, Hercules, CA, USA) and transferred to a nitrocellulose membrane using a Trans-Blot^®^ TurboTM blotting system (Bio-Rad, Hercules, CA, USA) with a pre-programmed protocol (2.5A, up to 25V, 3 min). Immunoblotting was performed using standard protocols: membranes were blocked for 1hr in 5% nonfat milk in TBS-T (0.1% Tween 20), then incubated for 1h in TBS-T with infected mouse serum (1:200) by using a Mini-Protean II multiscreen apparatus (Bio-Rad, Hercules, CA, USA), washed in TBS-T and then incubated for 1hr in TBS-T with an anti-Mouse IgG (Fab specific)–peroxidase antibody produced in goat (1:2,000, A2304, Millipore Sigma, St. Louis, MO, USA). Blots were imaged by chemiluminescent detection using Super Signal Pico chemiluminescent substrate kit (34080, Thermo Scientific, Rockford, IL, USA) on a ChemiDoc MP system (Bio-Rad, Hercules CA, USA).

### Mouse infection

Immune-competent RML mice are derived from a colony of Swiss-Webster mice established at NIH in 1935 and maintained at the Rocky Mountain Laboratories with an outbreeding program designed to intentionally maintain genetic diversity. RML mice (six to eight weeks old) were inoculated with 1 x 10^6^ cells in BSK-II (500 μl intraperitoneal and 250 μl sub-cutaneous) with *B*. *burgdorferi*, *B*. *afzelii* and *B*. *garinii* strains in triplicate. Six weeks post-infection, the mice were sacrificed and tissues (ankle joint, bladder, skin, and ear) were cultured in BSK-II supplemented with 50 μg/ml rifampicin, 2.5 μg/ml amphotericin B and 20 μg/ml phosphomycin (FD179, HiMedia, West Chester, PA, USA). Blood was collected for serology prior to inoculation by retro-orbital bleeding using a microhematocrit capillary and post-infection bleedings were performed by cardiac puncture.

### Ethics statement

Mouse infection studies were carried out in accordance with the Animal Welfare Act (AWA1990), the guidelines of the National Institutes of Health, Public Health Service Policy on Humane Care (PHS 2002) and Use of Laboratory Animals and the United States Institute of Laboratory Animal Resources, National Research Council, Guide for the Care and Use of Laboratory Animals. All animal work was done according to protocols approved by the Rocky Mountain Laboratories, NIAID, NIH Animal Care and Use Committee (Protocol Number 2014–021). The Rocky Mountain Laboratories are accredited by the International Association for Assessment and Accreditation of Laboratory Animal Care (AAALAC).

All infection studies were performed in an Animal Biosafety Level 2 facility according to protocols reviewed and approved by the RML Institutional Biosafety Committee and the RML IACUC. All work in this study adhered to the institution’s guidelines for animal husbandry, and followed the guidelines from the Guide for the care and use of laboratory animals. Animals were monitored at least once a day during the study. All efforts were made to minimize animal suffering. Mice were anesthetized by isoflurane inhalation prior to inoculation or blood withdrawal. Mice were euthanized by isoflurane inhalation followed by cervical dislocation. Mice showed no sign of illness and none of them died without euthanasia. A completed ARRIVE guidelines checklist is included in S1 Checklist.

## Supporting information

S1 Checklist Completed “The ARRIVE Guidelines Checklist” for reporting animal data in this study. (PDF)

S1 Table Genes of the chromosome found only in one or two strains: *B*. *burgdorferi* B31, *B*. *afzelii* BO23 and *B*. *garinii* CIP 103362. (XLSX)
